# Feeling unwell of passenger travel by small vehicles and associated risk factors in the North Shewa Zone, Oromiya, Ethiopia

**DOI:** 10.1186/s12889-024-19172-8

**Published:** 2024-06-24

**Authors:** Zelalem Tadese, Bayu Nesibu, Mesfin Sitotaw

**Affiliations:** 1 Department of Sociology, Salale University, Fitche, Oromiya Ethiopia; 2 Department of Education and Behavioral Study, Salale University, Fitche, Oromiya Ethiopia

**Keywords:** Feeling unwell, Passengers, Travel, Small vehicles, Associated risk factors

## Abstract

The current study investigated how and why sociocultural structures, situational conditions, and personal behavioural factors cause passengers to feel ill when travelling by minibuses, drawing on ideas from the social construction theory of illness. A significant objective was to investigate associated risk variables that influence passengers’ feelings of illness related to the social environment, addressing their beliefs, meanings, practices, and behaviours. A survey method was used to obtain data from 384 passengers for the study. The results of logistic regression indicated that feeling ill when travelling by minibuses differed from passenger to passenger; then, they had their own set of practical and emotional challenges that had no known medical reason. Compared with male and older passengers, female and younger passengers were more likely to feel ill. Furthermore, stress and role-set effects increased passengers’ experiences of feeling ill more than did passengers who had no stress prior to the trip and who had only one role. Additionally, passengers who travelled intermittently, utilized suppression techniques to lessen travel discomfort, and fastened seat belts were less likely to experience symptoms of illness. Passengers who travelled on unsafe roads and used alcohol before travel, on the other hand, were more likely to feel ill than those who travelled on safer roads and did not use alcohol before the trip. The findings suggest that passengers should be aware of predisposing conditions that result in illness, be able to rest before travelling, and use all suppressive methods to reduce or prevent illness while travelling by small buses.

**Supplementary Information:**

The online version contains supplementary material available at 10.1186/s12889-024-19172-8.

## Introduction

Much sociological research has documented how personal problems such as discomfort, dissatisfaction or illness in situations are linked to the influences of large-scale social forces, as guided by American sociologist Wright Mills’ theory of sociological imagination (1959). William C. Cockerham (2013), an American medical sociologist, hypothesizes that the social environment influences health and illness status and that particular social groupings or regions have greater health problems than others. Medicalization, as theorized by Peter Conrad (2007), emphasizes the fact that nonmedical problems caused by sociocultural factors are classified and treated as medical problems, frequently without strong proof of their medical nature. According to Conrad’s medicalization theory, feeling poorly, which is classified as illness, derives from practices, meanings, or perceptions associated with difficulties that are influenced by sociocultural forces. All theories, albeit in various ways, underline the ways in which macrosocial forces influence people experiencing illness, as theorized by social constructionism theory.

Motion sickness in general and feeling ill, in particular, while travelling in vehicles are venerable social and health problems [1]. Feeling unwell, on the other hand, is an illness that is defined as the way or pattern in which people perceive, interpret, and act in response to their personal troubles and discomforts owing to circumstances or situations [2]. Feeling unwell of passengers is specifically described by symptoms such as discomfort, terrible, or troubles as a result of vehicle travel in real situations. Symptoms also occur when individuals use simulators, movie theatres, or video games in virtual worlds [3, 4]. Furthermore, the symptoms of an illness that passengers develop while travelling by various sizes of vehicles include headache, dizziness, drowsiness, fatigue or nausea. Simply defined, an illness induced by sociocultural forces that affect individuals varies from one another [2]. Using this concept as a springboard, this study examined the risk factors for passengers feeling ill by presenting them in a social context.

A large percentage of the population has felt nauseous and uncomfortable at some point in their lives while travelling by automobile, boat, or airplane [5]. Feeling ill has remained a health issue for everyone at some point in their lives because it entails unfavourable events occurring while travelling by vehicle. According to a previous study, the organs of balance in the inner ears play an important role in determining the sense of unwellness of passengers while travelling by vehicle [4]. Deaf passengers, for example, were insensitive to motion sickness in general and felt ill, especially while travelling by vehicle.

Earlier studies focused more on vehicle energy efficiency and paid less attention to passenger comfort while travelling from one location to another. Transportation office and government control policies did not prioritize vehicle comfort-based strategies as a main focus [6]. Passengers in vehicles frequently experience health issues such as illnesses characterized by distress, malaise, and fatigue [6, 7]. Passengers feel ill or uncomfortable when travelling in various sizes of vehicles due to their perception of physical motion as well as what they experience about conditions or scenarios related to transportation [6, 8]. Furthermore, Mills’ sociological imagination contends that large-scale influences–societal (public) issues–have cognitively influenced people’ personal problems through general social standards, specific events, and personal experiences [9].

This study examined the relationship between illness and its associated risk factors, such as sociocultural structures, situational factors, and personal behavioral characteristics, in light of the probability that passengers may feel ill while traveling in small vehicles. In keeping with this premise, the following points were addressed in this study, which presented important research problems. The primary aim of this study was to investigate the risk factors for feeling ill as a result of travelling by small buses. Second, because a small bus was picked as a mode of transportation, some passengers complained about its discomfort in the study area. Since motion sickness and feeling illness is both health and social problems [1], this study examined why and how passengers felt ill, focusing on sociocultural fabrics, situational settings, and individual behaviour. To confirm methods that are useful for countermeasures in all-encompassing entities, this study investigated the multiple causal elements that cause passengers to feel ill. Furthermore, the purpose of this study was to identify the risk factors that contribute to passengers feeling ill while travelling by minibuses or small buses. Finally, the study investigated the factors that exposed passengers to feeling ill, as well as other issues that persisted when commuting in small vehicles.

According to the available literature, the feeling of illness among passengers while travelling by vehicle is induced by physical movement of the vehicle, driver disturbances, passive transport of passengers, and the travel behaviour or attitudes of passengers [10–13]. On the other hand, passengers’ lack of physical activity before travelling, the impracticality of alternate modes of transportation, and driving anxiety and disquiet naturally result in passengers feeling ill when travelling by vehicle [14–16]. Indeed, all of the research listed above highlighted the critical roles of linked risk factors for feeling ill; nevertheless, they did not address social aetiology as a relevant cause. They also lacked a rationale for linked risk factors based on societal attitudes that were used as motivations for exposing passengers to feeling ill before or during travel. Furthermore, the intervention methods by which such concerns should be minimized and passengers further assist in promoting well-being or health advantages prior to travelling by vehicle have remained unclear. Therefore, this study addressed all of the previously expressed questions to address these unanswered questions.

According to sensory conflict theory, passengers’ discomfort when travelling in vehicles is caused by inconsistent sensory inputs from their visual, vestibular, and proprioceptive systems with their previous sensory experiences [17]. Based on this theory, it was possible to argue that, in order to develop effective countermeasures, this study focused on the social milieu-related risk factors and the reasons why all passengers did not experience the same levels of fatigue, headaches, nausea, or dizziness even when they were traveling in the same small vehicles. Furthermore, exploring associated risk factors that result in feeling ill is an important beginning point for maximizing health benefits and demand, and interventions that help to minimize any discomfort linked with vehicle travel. With such gaps and scant or limited evidence, this study examined the relative effects of sociocultural structures, situational conditions, and individual behaviors on the feelings of passengers travelling by minibuses.

By contextualizing evidence from multilevel approaches, this study was able to investigate more effective programs to be executed by paying attention to evidence-based intervention tools that involve holistic and multidimensional inquiries by examining probable linked risk factors. This study contributes to understanding and adds insight to the body of knowledge about passengers’ feelings of illness as it is a medical problem caused by associated risk factors linked to sociocultural factors, situational conditions, environmental factors and personal behavioural factors. In addition, it requires treatment that is either based on the social, psychological or medical, in keeping with sociological imagination, social constructionism, and medicalization theories.

## Materials and methods

### Study setting and design

Between April 2022 and June 2022, six study sites were chosen for investigation at random in the community of the North Shewa Zone, Oromiya, from thirteen woreda and two town administrations: Warra Jarso, Kuyu, Degem, Fiche, Shararo, and Wachale. The North Shewa Zone is one of the zones of the Oromiya Regional State. This zone is located to the north of Ethiopia’s capital city, Addis Ababa (Finfinnee). In many sociodemographic features, the selected sites from woredas and town administrations are considered to have nearly similar characteristics. This study was conducted among passengers travelling by small vehicles. A small vehicle was operationally defined, according to information acquired from the Transport Office of North Shewa Zone, Oromiya, and the Circulated Directive for Transportation Service in 2020. Along with this point, a small vehicle was a small bus (minibus) with a carrying capacity of 12 to 24 passengers and was used to transport people from one location to another by travelling up to 150 km in a single journey.

Because it was difficult to find passengers who waited uniformly to go particular distances by minibuses or small buses at the study locations, the researchers were obliged to look for places where passengers were available. The researchers then communicated via phone the directors of all transport offices of the study area to confirm where people waited to travel. All office heads proposed bus stops or transit hubs as locations where people could be found. There was a bus station or travel centre serving the populace for transport service at each study site, with varying sizes of surroundings. Therefore, bus terminals or transport hubs were regarded as actual locations for passengers waiting in corridors as well as for investigators. To evaluate the contributing factors linked to passengers travelling by minibuses or small buses feeling ill, a community-based cross-sectional design was used.

### Inclusion and exclusion criteria

Male and female passengers who were waiting to board a minibus from a bus terminal or transport hub participated in the study. The current study included only passengers travelled by manual small buses of carrying capacity from 12 to 15. Furthermore, only passengers who did not have any condition or sickness that would normally result in them feeling unwell, as well as residents of the study area, were selected for the study. The study excluded young passengers under the age of 18, pregnant women, breastfeeding mothers, and women who had menstrual cycles throughout the examination, as well as those on medical treatment.

### Population and sample

The study was carried out on passengers who were chosen to travel by minibus at bus stations or travel hubs. Thus, the study population consisted of passengers waiting to travel by minibus in bus stations or travel hubs; the study samples consisted of passengers from various bus stops or travel hubs at each selected study site.

### Sample size determination and sampling

The sample size was calculated using a single population formula. Accordingly, the formula for the sample size determination used was: n = [p (1-p)] * [Z_α/2_)^2^/(e)^2^], where n denotes the sample size, Z_α/2_ is a critical value obtained from the standard normal distribution at the 5% level of significance and is equal to 1.96, and p represents a 50% proportion of experience feeling unwell among passengers who waited to travel by minibus in the bus station because there is no previous study on this topic and related to a 95% confidence coefficient. As a result, the method produced 384 sample sizes, which were determined by inserting numbers into the formula

$$\eqalign{{\rm{n}}\,{\rm{ = }} & {\rm{(0}}{\rm{.05*0}}{\rm{.5*1}}{\rm{.96*1}}{\rm{.96)/(0}}{\rm{.05*0}}{\rm{.05)}} \cr & {\rm{ = (0}}{\rm{.25*3}}{\rm{.8416)/0}}{\rm{.0025 = 384}}{\rm{.16.}} \cr}$$

The sample size was then dispersed in equal amounts to each study site. Each study site received a sample size of 64 based on the computation.

A multistage sampling method was employed. Using primary sample units, a random sampling technique was used to pick six research sites from the fifteen identified study sites because all of the study sites were considered to have comparable characteristics in terms of employing a minibus as a mode of transportation. Passengers were randomly picked at each bus station in the secondary sampling units based on the aforementioned criteria. Each study site received a proportional allocation of the sample size. Finally, the assigned sample size was reached by applying a simple random sampling procedure.

### Data collection methods

The survey was carried out by adapting questions from the Motion Sickness Severity Scale (MSSS) instrument, which was created for assessing symptoms of motion sickness in general, and feeling unwell in particular, that passengers experienced while travelling in a variety of vehicle sizes in a real environment. The instrument measured self-reported symptoms (illness) associated with vehicle travel and recommended the creation and study of countermeasures. Furthermore, the instrument queried associated risk factors for passengers feeling ill when travelling in various sizes of vehicles as a mode of transportation service, and provided quick and accurate measurements of travel-related symptom intensity [18].

Therefore, the study included 5 item questions emphasizing sociodemographic measurements, 3 item questions compiling general information about passengers feeling ill, 5 item questions adapted for sociocultural measurements, 8 item questions emphasizing situational measurements, and 4 item questions emphasizing individual behavioural measurements. As an interview schedule tool, all of the included and adapted questions were combined into a single form. An interview schedule is a thoroughly and logically designed tool consisting of many questions that investigators employ to acquire primary data from study participants by asking them because it is difficult to distribute and collect tool back. Following the development of an interview schedule tool in English, it was translated into Afaan Oromo and Amharic, the two languages spoken at each study site. Trained data collectors were recruited and then provided rigorous training on the study’s objectives and instrument questions.

### Dependent variable and independent variables

The outcome or dependent variable of this study was passengers feeling ill while riding on a minibus. Symptoms such as nausea, headache, fatigue, and dizziness were used to assess passengers who were feeling ill. A passenger who experienced at least one of the symptoms listed above while travelling by minibus is said to feel ill. If a passenger has any of the symptoms listed above while travelling by minibus, they are said to feel ill. As a result, the outcome variable, passengers feeling ill while travelling by minibus, was divided into binary responses and specified as follows:

$$Y = \left\{ \matrix{0\quad {\rm{if}}\,{\rm{pasengers}}\,{\rm{had}}\,{\rm{not}}\,{\rm{experienced}}\,{\rm{atleast}}\, \hfill \cr \,\,\,\,\,\,\,{\rm{one}}\,{\rm{of}}\,{\rm{the}}\,{\rm{symptoms}}\,{\rm{for}}\,{\rm{past}}\,{\rm{3}}\,{\rm{months}} \hfill \cr 1\quad {\rm{if}}\,{\rm{passengers}}\,{\rm{had}}\,{\rm{experienced}}\,{\rm{atleast}} \hfill \cr \,\,\,\,\,\,\,{\rm{one}}\,{\rm{of}}\,{\rm{the}}\,{\rm{symptoms}}\,{\rm{for}}\,{\rm{past}}\,{\rm{3}}\,{\rm{months}} \hfill \cr} \right.$$

The study assessed whether passengers felt ill while riding on a minibus if the four symptoms listed above met the following criteria. First, with nausea, passengers who feel ill while travelling are measured if they suffer situations that cause them to lose the ability to taste and detect an unsettled stomach. When study participants were unhappy, had problems, or were worried about their journeys while travelling, they were more likely experience headaches. If study participants behaved in ways that generated discomfort and then added to irritations during travel, they felt ill. Third, if the study participants experienced physical or mental exhaustion while travelling and believed that they would be unable to continue travelling by minibus, they experienced fatigue symptoms. Finally, due to dizziness, the study participants experienced feeling unwell if they had lost their balance, felt difficulty sitting steadily, or experienced a reeled sensation in the head with a tendency to tumble when travelling. Thus, the study analyzed the probabilities of symptoms that passengers developed or felt while travelling by minibus by executing a forced answer, yes or no choice of the previous three months of an investigation. Participants in the study who replied yes were judged to be ill while travelling by minibus. Those who said no were assigned to groups without feeling ill.

The following independent variables were evaluated as associated risk factors for passengers feeling ill when travelling by minibus after a comprehensive evaluation of previously available research that was referenced as sources by this manuscript. The sociodemographic characteristics of the study participants (age, sex, educational level, income, occupation), general information about passengers feeling unwell (past experiences of travel by minibus, feeling unwell while travelling or not, symptoms for passengers feeling unwell), sociocultural measurements (stress or not, traveling by minibus before or not, traveling with worries about unlawful acts or not, lack of awareness or not, role set effects or not), situational measurements (minibus speed fear or not, less travel by minibus or not, working long hours before travel or not, road unsafety or not, lack of center for information about illness or not, inability to suppress ride discomfort or not, long distance travel or not, lack of seat belt use or not), and individual behavioral measurements (eating poorly or not, failing to physical exercise before travel or not, alcohol use before travel or not, quarreling in minibus during travel or not) were treated as independent variables.

### Statistical analysis

The distribution of the study participants presented using descriptive statistics such as frequencies and percentages. A binary logistic regression model with odds ratios at the 95% confidence level was used to examine the estimated effects of the independent variables on passengers feeling ill when travelling by minibuses. To evaluate the statistical performance of continuous predictor variables intended to classify their efficacy discrimination, the receiver operating characteristics (ROC) curve analysis was adapted. SPSS software version 20 was used to analyze the data. The Pearson chi-square test was used in conjunction with bivariate statistical analysis to investigate the association between each independent variable and passengers’ feelings of ill-health while riding in a minibus. Then, in the multivariate logistic regression model, all significant independent variables with a p-value less than 0.25 in the bivariate analysis were included. Furthermore, the variance inflation factor (VIF) is used to determine whether independent variables are multicollinearity. The alpha level used for all the statistical tests was 0.05.

## Results and discussions

### Sociodemographic characteristics of the study participants

A total of 384 passengers took the survey and replied to the information supplied. There were 239 (62.2%) male passengers and 145 (37.8%) female passengers. Furthermore, 56 (14.6%) of the passengers in the survey were under the age of 27, on the other hand 133 (34.6%) were 28 to 37 years old, 89 (23.2%) were 38 to 47 years old, and 76 (19.8%) were 48 to 57 years old. In the end, 30 (7.8%) of the passengers were over the age of 55.

In terms of occupation, 53 (13.8%) of the passengers in the survey were high school students and above, while 121 (31.5%) were unemployed at the time of the study. Furthermore, 73 (19%) of the individuals who participated in the study were passengers working for the government or nongovernmental organizations, and 137 (35.7%) were commerce passengers. In terms of schools attended, 91 (23.7%) and 94 (24.5%) passengers who participated in the study attended primary and elementary schools, respectively, whereas 86 (22.4%) of them attended high schools and 113 (29.4%) attended college and above schools. Finally, 43 (11.2%) of those who participated in the study earned less than 2,000 Ethiopian Birr per month, 52 (13.5%) earned a monthly income of 2,001 to 4,000 ETB per month, and 60 (15.6%) earned a monthly income of 4,001 to 6,000 Ethiopian Birr. Table 1 shows the other details of the passengers’ monthly income.

**Table 1:** Sociodemographic characteristics of the study participants in 2022

Variables	Categories	f	%
Sex	Female Male	145 239	37.8 62.2
Age (in year)	< 27 28–37 38–47 48–57 > 57	56 133 89 76 30	14.6 34.6 23.2 19.8 7.8
Occupation	Student Unemployed Employee Commerce	53 121 73 137	13.8 31.5 19 35.7
Educational Level	Primary school (1–4) Elementary school (5–8) High schools (9–12) College and above	91 94 86 113	23.7 24.5 22.4 29.4
Income per month (Ethiopian Birr)	< 2000 2,001–4,000 4,001–6,000 6,001–8,000 8,001–10,000 > 10,000	43 52 60 73 77 79	11.2 13.5 15.6 19 20.1 20.6
	**Total**	**384**	**100**

Source: Authors Survey, 2022

### Minibus measurements of passengers feeling unwell during travel

The overall measurement of the dependent variable, passengers feeling ill while travelling by minibus, was based on the symptoms of headaches, dizziness, fatigue, and nausea. Among the passengers who travelled by minibus and took part in the study, 123 (32%) felt ill, whereas 261 (68%) did not experience any of the symptoms listed above. Among the passengers who became ill while travelling by minibus, 31 (8.1%) experienced headaches, while 27 (7%) experienced dizziness. Furthermore, due to fatigue and nausea symptoms, 22 (5.7%) and 43 (11.2%), respectively passengers felt ill while travelling by minibuses. Table 2 displays all of the reported statistics.

**Table 2:** Measurements for passengers feeling unwell during travel by minibus, 2022

Variables	Categories	f	%
Feeling unwell while travelling by minibus.	Yes No	123 261	32 68
Measurements of passengers feeling unwell	Headaches Dizziness Fatigue Nausea	31 27 22 43	8.1 7 5.7 11.2

Source: Authors Survey, 2022

### A bivariate analysis of risk factors and passengers feeling unwell

A bivariate analysis was performed to investigate the association between each risk factor and the outcome variable of passengers feeling ill when travelling by minibus. According to the findings, independent variables such as sex, age, stress, traveling with concerns about illegal acts, role-set effects, less travel by minibuses, using measures to reduce ride discomfort, unsafe road transportation, working fewer hours, seat belt use, long distance travel, and alcohol use before travel were significantly associated with the outcome variable at p-values less than 0.25. Along these lines, using multivariate logistic regression analysis, all of the significant independent factors in the bivariate analysis were analyzed for additional investigation to assess their impacts on the outcome variables, passengers feeling ill. Table 3 summarizes the findings of the analysis.

**Table 3:** Multivariate analysis of variables/covariates predicting feeling unwell of passenger travel by minibus, 2022

Variables with respective categories	Feeling unwell of passengers travel by minibus		COR (95% CI)	AOR (95% CI)
	Yes	No		
Sociodemographic variables	f (%)	f (%)		
**Sex** Male Female	37(9.6) 86(22.4)	202(52.6) 59(15.4)	0.02(0.01,0.06)*	0.34(0.25,0.43)**
**Age (year)**	-	-	0.15(0.01,0.05)^*^	0.25(0.10,0.64)^**^
**Income per month, (Ethiopian Birr), in Scale**	-	-	-0.04(-0.08, 0.03)	1.06(0.45,2.51)
**Occupation** Student Unemployed Employee Merchant	43(11.2) 37(9.6) 24(6.3) 19(4.9)	10(2.6) 84(21.9) 49(12.8) 118(30.7)	-0.06(-0.11,-0.02)^*^	0.61(0.24,1.55)
**Educational Level** Primary school (1–4) Elementary schools (5–8) High schools (9–12) College and above	25(6.5) 21(5.5) 36(9.4) 41(10.6)	66(17.2) 73(19) 50(13) 72(18.8)	0.02(0.01,0.04)	1.02(0.98,1.07)
**Socio-Cultural Factors (yes)**				
Absence of experience with minibus travel	26(6.8)	358(93.2)	0.02(-0.06,0.08)	0.61(0.24,1.55)
Lack of awareness about illness on vehicles	34(8.9)	350(91.1)	1.08(0.43,2.70)	0.46(0.15,1.47)
Stress	98(25.5)	286(74.5)	0.13(0.03,0.24)^*^	5.99(2.06,17.46)^**^
Role-set effects	101(26.3)	283(73.6)	0.24(0.14,0.32)^*^	1.40(1.05,1.87)^**^
Travelling with worries for unlawful acts	48(12.5)	336(87.5)	0.14(0.04,0.23)^*^	0.61(0.07,5.10)
**Situational Associated Factors (yes)**				
Minibus speed fear	19(4.9)	365(95.1)	0.01(-0.09,0.10)	0.55(0.12,2.52)
Travel by minibus on rare occasions.	70(18.2)	314(81.8)	0.08(0.02,0.10)^*^	1.33(0.50,3.51)
Lack of center for information about Illness	22(5.7)	362(94.3)	0.03(-0.08,0.14)	1.57(0.48,5.12)
Applying measures for suppressing ride discomfort	83(21.6)	301(78.4)	0.49(0.37,0.53)^*^	0.06(0.01,0.69)^**^
Unsafe road transport	97(25.3)	287(74.7)	0.02(0.01,0.26)^*^	1.36(1.15, 1.61)^**^
Intermittent distance travel	49(12.8)	335(87.2)	0.38(0.29,047)^*^	0.36(0.13,0.99)^**^
Seat belt use while travelling	52(13.5)	332(86.5)	0.08(0.01,0.10)^*^	0.36(0.14,0.89)^**^
Working less hours before travel	41(10.7)	343(89.3)	0.02(0.06,0.11)*	0.88(0.39,1.95)
**Individual Behavioral Factors (yes)**				
Alcohol use before travel	87(22.7)	297(77.3)	0.02(0.01,0.03)^*^	9.62(4.69,19.76)^**^
Eating poorly	23(6)	361(94)	-0.03(-0.14,0.08)	1.15(0.59,2.26)
Failing to physical exercise before travel	15(3.9)	369(96.1)	-0.04(-0.15,0.06)	0.94(0.38,2.32)
Quarrelling in minibus during travel	29(7.6)	355(92.4)	-0.01(-0.05,0.04)	1.28(0.62,2.64)

*significant at *p* < 0.25 ** significant at *p* < 0.05

COR: Crude Odds Ratio, AOR: Adjusted Odds Ratio

### Goodness of fit of the model

The Hosmer and Lemeshow test with chi-square statistics (χ^2^ = 6.286, p-value = 0.615) and likelihood ratio test with chi-square statistics (χ^2^ = 219.817, p-value < 0.001) confirmed that the logistic regression model used for the multivariate analysis was sufficiently fitted to the data. A 1.244 was the Pearson chi-square statistics to degrees of freedom ratio. The fact that the ratio was close to 1 suggested that the logit model was well suited to the data. Thus, utilizing the data fitted to the model and all of the provided values, a logistic regression model for multivariate analysis can be built.

### ROC curve analysis

**Fig. 1:**
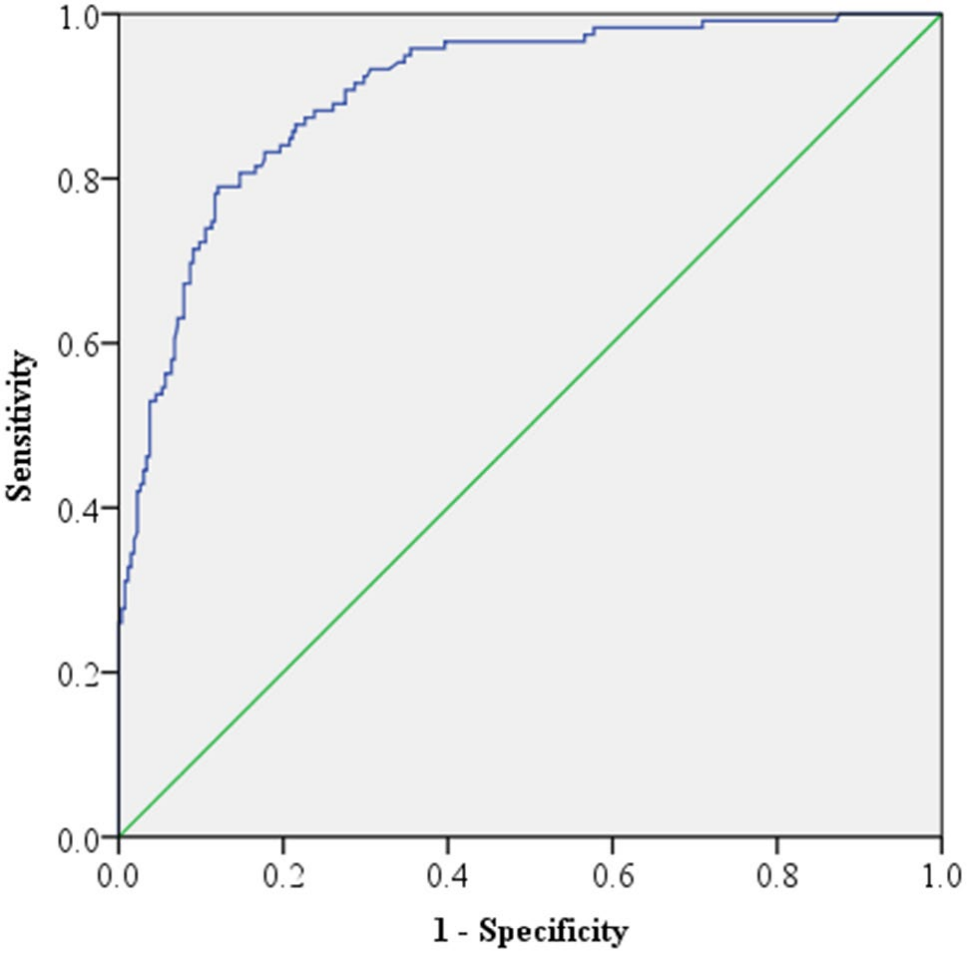
ROC Curve Analysis

The performance of continuous predictor variables intended to categorize predictor efficacy discrimination was assessed statistically using the ROC curve analysis. A diagonal reference line was used to measure the area under the curve (AUC) with a cutting-off point of 0.5 in order to assess the discrimination efficacy for the identified risk factors related to passengers’ feelings of illness while travelling in minibuses. The identified associated risk factors had stronger discriminating efficacy because the AUC was closer to 1 or the curve was closer to the upper left corner, which indicated better efficacy and accuracy of predicators, as seen in Fig. 1. According to the results of the analysis presented in the classification table, sensitivity was 0.875 and specificity was 0.766.

### Associated risk factors for passengers feeling unwell

Table 3 shows the findings of a multivariate analysis of variables that predict passengers feeling ill while travelling by minibus. According to the findings, sociodemographic variables such as gender and age of those polled had a substantial impact on passengers feeling ill while traveling by minibus. In other words, male passengers were 0.34 times less likely than female passengers to feel ill or feel unwell while traveling by minibuses (AOR: 0.34; 95% CI: 0.25 to 0.43). On the other hand, as passenger age increases by one unit, the probabilities of acting in reaction to personal problems or travel discomfort decreases by 0.25 times (AOR: 0.25; 95% CI: 0.10 to 0.64).

The investigation revealed that sociocultural factors had a substantial impact on experiencing travel-related symptoms, which might be interpreted as an indication of feeling ill when passengers travelled by minibuses. Passengers who experienced stress before travelling by minibuses, for example, were 5.99 times more likely to experience trip discomfort than passengers who did not experience stress before travelling (AOR: 5.99; 95% CI: 2.06 to 17.46). Another aspect that characterized the passengers’ condition and whether they felt ill while riding on a minibus was role-set effects. Passengers with a role-set were 1.40 times more likely than those without a role-set to feel ill (AOR: 1.40; 95% CI: 1.05 to 1.87).

Situational associated factors, also called contextual factors, are the conditions or circumstances surrounding passengers’ feeling ill while they travel in minibuses. These factors can influence or impact passengers’ behavior or outcome with regard to their health within that particular travel scenario. Situational factors were considered while travelling by minibuses to assess whether passengers experienced symptoms that were subjectively characterized as suggesting illness while travelling. According to the research given in Table 3, passengers who took activities to suppress ride pain or feel unwell while travelling by minibus were 0.06 times less likely to suffer travel problems than those who did not take suppressive measures for ride discomfort (AOR: 0.06; 95% CI: 0.01 to 0.69). Furthermore, not only did the suppression action influence whether passengers interpreted feeling ill, but so did unsafe (risky) road transport. Passengers travelling on minibuses on unsafe roads were 1.36 times more likely to feel ill than passengers travelling on safer roads (AOR: 1.36; 95% CI: 1.15 to 1.61).

Alternatively, passengers who travelled a specific distance by minibus intermittently (sporadically) were 0.36 times less likely to feel ill than those who travelled continuously (AOR: 0.36; 95% CI: 0.13 to 0.99). A safety device, such as a seat belt, can, on the other hand, impact passengers’ feelings of discomfort while travelling by vehicles from one location to another. For example, passengers who travelled by minibuses while buckling seat belts were 0.36 times less likely to become unwell than those who did not (AOR: 0.36; 95% CI: 0.14 to 0.89).

A personal behavioural component determined whether passengers felt ill when traveling by minibus. The study revealed that passengers who used alcohol before travelling were 9.62 times more likely to experience symptoms of feeling ill while traveling than were those who did not use alcohol before travelling (AOR: 9.62; 95% CI: 4.69 to 19.76).

### Discussion

There is a scarce of research on the social context in search of social forces and patterns that influence why and how people see, understand, and act in reaction to illness [2, 9]. In addition, passengers may encounter a condition or circumstance that causes them to feel hostile, which is described as an illness while travelling by vehicle [7]. Some studies have analyzed passengers who feel ill in simulated surroundings and real environments, by combining various sizes of vehicles.

Personal problems, as defined by American sociologist Wright Mills in 1959, are a predicament linked to the cultural norms, traditions, and values that constitute social forces. Feeling ill while travelling is considered one of people’s personal problems. Thus, this study applied theory to investigate how and why sociocultural forces influence passengers’ feeling of illness while commuting by minibuses. Furthermore, as theorized by Peter Conrad (2007), medicalization involves illness caused by people’s practices and perceptions of situations, resulting in unpleasant or uncomfortable outcomes. According to this viewpoint, people’s attitudes, thoughts, emotions, beliefs, actions, or perceptions are nonmedical situations that cause ill-health and must be medicalized in human life. Based on this viewpoint, this study examined situational aspects that contributed to passengers feeling ill when commuting by minibus.

Furthermore, as amended by Sigrun Olafsdottir (2013), social construction theory focuses on how people think about ills, what people view as reasons for ills, and how all of this is shaped by cultural and social forces. Along these lines, this study attempted to examine individual behavioural traits that contributed to minibus passengers feeling ill. Finally, according to William C. Cockerham (2013), an American medical sociologist, the social environment can cause inequities in health and illness based on gender, class, ethnicity, and occupation. Such disparities in health or illness are socially patterned issues that must be investigated by examining the underlying risk variables with social patterns and contexts. This study derived four key themes from the surveyed data using ideas from the abovementioned theories: (a) feeling unwell and sociodemographic variables, (b) sociocultural factors and feeling unwell, (c) the influences of situational factors on feeling unwell, and (d) the effects of individual behavioural factors on feeling unwell.

#### Findings in relation to understanding passengers feeling unwell

According to the study’s findings, 32% of passengers in the study area felt unwell when riding on a minibus. To poll passengers who were feeling ill, many factors with well-defined groups were used. The most common symptom suggested by passengers who felt ill when traveling by minibus was nausea (11.2%), followed by headache (8.1%). Other symptoms associated with feeling ill included dizziness (7%) and fatigue (5.7%).

The findings supported William C. Cockerham’s (2013) theory of social environment imperatives for affecting people’s behaviours, attitudes, expectations, perceptions, roles, and life outcomes unequally as acceptable measurements for members of either sex. Gender disparities in social relationships in tapestries have caused inconsistency between males and females who feel illness while travelling by minibuses. In line with prior research [7, 15, 19], this study revealed that female passengers felt more ill than male passengers. All of the research listed above did not precisely define the categories of vehicles, whether small or large. Furthermore, health issues encountered by passengers while travelling were not expressly stated as sickness or illness, but were just described as motion sickness. The current study, on the other hand, described vehicle type as manual and recognized health concerns associated with travel as feeling unwell, which is regarded as an illness.

Passengers who feel ill typically exhibit symptoms that can only be confirmed by subjective interpretation and are not recognized as having disease or sickness by other people, including health professionals. In contrast, sickness is a health problem that is described by the reaction of others or is medically diagnosed by health professionals based on the manifested symptoms [9]. In other words, the social constructionism theory looks at how cultural and social processes impact health and the meaning and experience of illness. Feeling ill is a social notion that includes, but is not limited to, people’s behaviours, expectations, perceptions, and the role they play in their social environment [2]. Given these critical issues, the ways in which females work, believe, and act in society before travelling may contribute to creating a sense of ill-health. In other words, female passengers may have travelled less than male passengers as a result of such behaviours, expectations, or roles. All of the aforementioned effects were deemed risk factors and may contribute to feeling ill. Female passengers’ employment, societal beliefs about them, and tapestry involvement in activities prior to travel may cause them to experience problems that cause them to feel ill while travelling. Passengers feel ill as a result of their social conditions, as suggested by Wright Mills’ sociological imagination theory.

Another factor that contributed to passengers feeling ill was the number of years they had lived. The findings revealed that younger passengers were more susceptible to feeling ill than older passengers while travelling by minibuses. The findings were consistent with prior research [4, 10, 20–22] which was conducted at diverse locations and times. According to William C. Cockerham (2013), our social environment does not totally define our behaviours, attitudes, and life outcomes, but it shapes or reshapes them. As a result, as people becoming aged, they are considerably more prone to developing a variety of health issues, including chronic disease and other disorders. Consequently, they spent more time practicing preventive measures or seeking health behaviours, and such efforts boosted their chances of keeping healthier than younger persons did [9]. Along these lines, older passengers may suffer from a variety of illnesses throughout their lives, including while travelling by minibuses in particular and vehicles in general. As a result, encountering such health issues drove or dragged them to implement preventive measures as countermeasures to what they witnessed. Furthermore, older passengers may have travelled vast distances, which allowed them to adjust and find a way out of the challenges encountered while traveling by minibus. As a result, older passengers may have had to deal with additional health issues, such as feeling ill while travelling. Because younger travellers experience fewer complications than older passengers across life phases, they should take adequate and suitable actions to reduce their disease burden prior to or during minibus travel.

#### Findings in relation to socio-cultural factors and feeling unwell

Stress contributed passengers to feel ill while travelling by minibus. Stress is an emotional and mental tension caused by extremely negative social settings or situations, and those who are in such situations experience health problems such as illness [9]. According to sociological imagination theory, social stress is a negative social circumstance that is at the base of personal difficulty, such as feeling ill as a result of vehicle motion. Passengers who were under stress before the trip reported feeling ill when travelling by minibus. In agreement with the hypothesis expressed in earlier studies [3, 20, 23, 24], the findings analyzed the relationship between stress and passengers feeling ill. Nonetheless, previous investigations did not adequately explain the patterns and nature of stress which is recognized as determinant of travel. According to Peggy A. Thoits (2010), a medical sociologist, stress is a social condition that influences people who are confronting despair and terrible situations. Furthermore, social stress is a tension caused by undesirable social settings such as negative life occurrences, tensions, strains, and accidents [2]. The study revealed that social stress that endured while travelling by minibuses caused passengers to feel ill. In other words, feeling ill when traveling by minibus was caused by the social stress that passengers experienced before. The stressors that contributed to feeling ill could have included the impracticality of good health behaviours, involvement in risky health behaviours, physical inactivity, and decreased supporting social networks. While Wright Mills dealt with sociological imagination, he contended that macro level issues cognitively influence people’s particular problems as they pass through a general societal standard and, more specifically, personal experiences. Having a sense of control over one’s life and improving one’s self-esteem in the face of poor social settings, can buffer the effects of stress.

Role-set effects are another sociocultural element connected with passengers feeling ill. The role-set impact was a significant variable in explaining how and why individuals felt ill while travelling. This study is a continuation of earlier study undertaken in the same research area [7]. Merton (1957) defines a role as “issues such as duties, responsibilities, behaviours, or expectations that a person occupies in society owing to them either through ascribed or achieved efforts.” Furthermore, a role-set is an array of roles that are associated with a specific rank. According to a stated theory, role-set impacts were described as having several responsibilities, being busy with various tasks, working long hours, and obtaining less rest as a result of activities or tasks. As a result, passengers in a variety of roles may have felt more ill while travelling by minibus. Wright Mills defined personal trouble as “feeling unwell,” and stated that communities frequently assign such trouble to the fault of individual experiences, but that the impacts of structural problems frequently underpin personal trouble. Such duties may cause passengers to feel ill while travelling by minibus. Passengers with several duties should decrease tasks or obtain adequate rest before travelling by minibuses.

#### Findings in relation to the influences of situational factors and feeling unwell

Travel discomfort suppression methods are steps that can be taken to lessen or prevent people from feeling ill because of exposure while traveling in vehicles [25, 26]. The findings demonstrated that suppressing predisposing factors that are thought to induce ride discomfort will support passengers in feeling comfortable while travelling by minibuses. These findings are consistent with those of prior studies conducted by scholars in various locations and at various times [20, 26, 27]. All of the research described above advised countermeasures that could be viewed as model-based motion control systems for transportation authorities, but they did not focus on what and how suppression actions were performed by placing them in the social context. Furthermore, no efforts have been made identify situations and behaviours that can be deemed social problems but are characterized as medical problems via the prism of medicalization theory. There was no single superior countermeasure proposed against feeling ill while travelling by vehicle. Furthermore, none of the studies listed above predicted the actions for countermeasures that may be used to inhibit feeling unwell. Passengers who felt ill while travelling in vehicles were subdued utilizing specific types of acts deemed countermeasures [7]. However, these studies did not adequately explain what passengers used for suppression when travelling by vehicle. Because they felt ill while travelling, they may have eaten gum, slept with their neck down, opened windows to allow free air to circulate in and out, and employed lemon or orange as suppressing devices. Passengers who have tried all of the above tactics and still have not found a solution may elect to travel a set distance intermittently until they reach their destination.

Given the risks of unsafe road transport in terms of motion discomfort, the findings revealed that passengers travelling by minibuses felt ill. These findings were consistent with those of previous studies that addressed precarious road transport-related health problems only from the perspective of road nature [22, 23, 28, 29]. Even if the studies did not classify and treat them as medical concerns in terms of illness or disorders from a medicalization standpoint. Unsafe road transport is defined as a voyage in which passengers and drivers do not take the essential steps to protect themselves against road traffic accidents/injuries, motion sickness, and illness. Unsafe road transportation includes difficulties such as the curving shape of road types, vehicle speed, travelling in close quarters with other passengers, and travelling beyond permitted hours. Passengers feeling ill may have resulted from either of the aforementioned causes. All of these factors were raised as medical concerns for people who were ill while traveling by minibus. Finally, a road safety policy aimed at lowering health concerns caused by vehicle motion while travelling would advise making road public transport more appealing to travelers. Both the government and the road authority must devise new tactics or collaborate to coordinate approaches to make public road transit safer and to increase public trust.

Intermittent travel by minibuses proved more effective than continuous travel in reducing unsatisfactory situations caused by road transportation. The findings were consistent with previous investigations conducted in different locations [30, 31]. These studies primarily addressed passengers who travelled small distances, stating that as the number of kilometers travelled decreased, so did the likelihood of fronting difficulties caused by vehicle motion. Nonetheless, they did not describe the situations or conditions under which voyagers travelled from one location to another. Furthermore, they did not put forth enough effort to understand how such nonmedical behaviours and experiences become medical problems that require additional therapy. The findings revealed that intermittent travel helped passengers feel less ill while travelling by minibuses. Intermittent travel is a journey that comprises travelling for a set distance, resting, and then continuing on. In other words, it is a voyage characterized by getting out of the minibus to temporarily halt travel discomfort and then resuming the journey until reaching the destination. Such a journey may be categorically stated as having considerable short distance travel with an increased tariff until arriving at the destination location and may aid in reducing travel pain. Passengers who felt ill while riding in a minibus were advised to employ such tactics to alleviate their suffering.

This study revealed that buckling a seat belt while travelling, in addition to protecting against an accident and being thrown out of the seat, helped to reduce feelings of illness. The findings coincided with earlier research conducted in various locations [11, 32, 33]. All of the mentioned above studies showed the importance of seat belt use only in terms of accident and injury prevention. A previous study [27] conducted in another place concluded that utilizing a seat belt or safety belt is very important for human safety because it decreases the severity of illness induced by vehicle motion. However, none of the studies listed above focused on buckling seat belts while travelling in vehicles, and not fastening seat belts affects many people. Furthermore, the abovementioned studies did not define how seat belt use could be useful in preventing passengers from becoming ill. Seat belt use, for example, may assist passengers in becoming more stable and safer while travelling by minibuses against acceleration, braking effects, and discomfort from transportation road up and down. As a result, such systems only worked in vehicles that had seat belts. Thus, relevant bodies and traffic authorities have proposed monitoring whether passengers utilize safety belts and vehicles equipped with passenger safety belt facilities.

#### Finding in relation to individual behavioral factors and feeling unwell

The amount of alcohol used by passengers prior to travelling by minibus was significant in causing them to feel ill. This study’s findings were consistent with those of earlier studies [7, 13]. Previous research investigated health-related concerns while travelling in vehicles that were also caused by alcohol use behaviours prior to travel. Other studies have shown that drinking alcohol before a trip causes passengers to feel ill when travelling [11, 12, 30]. It was easy to understand that not all passengers felt ill as a result of travelling in automobiles whose drivers had used alcohol before the trip. In turn, it hints at the social construction theory, which states that certain social realities exist only because individuals decide they do, and that these realities, as a result, have no objective basis. After a heavy meal, alcohol use is defined as the consumption of beer and its products, as well as native beverage items such as *Tella* and *Arake* (*Katikal*), for recreational and digestion purposes. Because alcohol is a depressive and psychotropic chemical that decreases the functioning of the central nervous system, if drivers or passengers use alcohol before traveling, their expectations and perceptions of the trip may change. Furthermore, drivers may accelerate the minibus beyond the permitted speed, causing passengers to perceive or experience discomfort as a result of the acceleration. High levels of vehicle acceleration are projected to increase the risk of motion sickness, a major hindrance to driving comfort [34]. Passengers may experience restlessness and fear, resulting in headaches when travelling. According to the findings of this study, passengers and drivers who feel ill due to alcohol use before travelling should avoid doing so.

#### Limitations and directions for future research

Certain design elements and methodological choices may have influenced the findings of the present study. Because the data were cross-sectional, causal inference may be difficult. It is also likely that access to other risk factors by placing them in a social environment that possibly includes critical variables would have strengthened the study and enhanced the full potential of the insights. Furthermore, sociodemographic features of the passengers in the study area reflected those passengers who waited for small buses at bus terminals rather than how and from where they came to either rural or urban areas. It is thus possible that the linked risk factors evaluated could serve as a proxy for other passengers waiting for transportation in a public area or roadway. However, this study aimed to determine the influences of sociocultural elements on health concerns associated with vehicle travel by specifying vehicle size, although the current study included only small buses. Furthermore, because the transportation system in the study area permitted passengers to select vehicles, small buses were not the preferred mode of transportation for all passengers in the study area. It should be noted, however, that the current study may have overstated the influences of social forces by not including the small bus as a means of transportation for all passengers in the analysis.

Another limitation was linked risk causation modelling of feeling ill among passengers, which was effective for understanding illness related to travel but could not explain the amount of severity that passengers experienced. The logistic regression statistical tools and evaluation of associated risk factors have limited power to explain why and how passengers become ill this year rather than last. As a result, for a firm recommendation, a long-term study is needed. Exploring other components of social, contextual, and socioeconomic advantage beyond those studied here may thus contribute to a better understanding. More research into the shared determinants of feeling ill is needed, and this study was one attempt. Finally, additional research and studies in the future are advised to look at the factors that lead to the development of motion sickness in general and the experience of illness in particular by concentrating on specific risk factors that are either psychological, environmental, or societal in nature.

## Conclusions

The current investigation revealed that related risk variables have the potential to make minibus passengers feel ill. As a result, the sociodemographic features of passengers, such as being female or younger, had a greater impact on feeling ill while travelling by minibuses. Furthermore, sociocultural factors such as stress before travel and being responsible for a variety of jobs contributed to passengers feeling ill while travelling by minibus. In terms of the effects of situational circumstances, not taking measures to alleviate pain and risky road transportation caused passengers to feel ill while travelling by minibus. In contrast, feeling or interpreting illness was less common on trips where participants travelled sporadically and utilized a seat belt or safety belt. Finally, drinking alcohol before traveling with passengers and drivers had a more direct effect on the link between feeling ill and travelling by minibus.

### Electronic supplementary material

Below is the link to the electronic supplementary material.

Supplementary Material 1

## Data Availability

The data sets used and/or analyzed during the current study are available from the corresponding author upon a reasonable request.
