# Supplementary material for: Feeling unwell of passenger travel by small vehicles and associated risk factors in the North Shewa Zone, Oromiya, Ethiopia
Source: BMC Public Health. 2024 Jun 24;24:1672. doi: 10.1186/s12889-024-19172-8 (PMC11194939; doi:10.1186/s12889-024-19172-8)
Supplement: Supplementary file 1 — Supplementary Material 1 [file 12889_2024_19172_MOESM1_ESM.docx]

**Salale University**

**College of Social Sciences and Humanities**

**An Interview Schedule for Passengers in the North Shewa Zone, Oromiya**

A team from interdisciplinary fields at the College are currently conducting a research entitled “**Feeling Unwell of Passenger Travel by Small Vehicles and Associated Risk Factors in the North Shewa Zone, Oromiya, Ethiopia.”** Your thoughtful and heartfelt responses to the questions below greatly contribute to the success of our research. As a result, we respectfully ask that you provide an honest response that will remain private. You do not have to write your name when answering the questions, but by signing below, you are granting us a written informed consent to use your anonymized data for publication and distribution and to participate in this study.

| **Sociodemographic Variables** | | | **Code** |
| --- | --- | --- | --- |
| 1 | Sex of the respondents: | 1=Female 2= Male |  |
| 2 | Age of respondents: | ________________ |  |
| 3 | Education Level |  |  |
| 4 | Income per month |  |  |
| 5 | Occupation of respondents: | _______________ |  |
| **General Information about Feeling Unwell and Vehicles** | | | |
| 6 | Have you used a small vehicle for travelling? | 1. Yes 2. No |  |
| 7 | Per a week how many times have you traveled by small vehicle? | ____________________________ |  |
| 8 | In the past three months, have you experienced any symptoms while you travelled by small vehicle? | 1. Yes 2. No |  |
| 9 | If **yes**, what type of symptom have you experienced? | 1. Dizziness 2. Fatigue 3. Headache 4. Nausea |  |
| **Socio-Cultural Associated Factors** | | | |
| 10 | Have you any stress that made you experience a symptom? | 1. Yes 2. No |  |
| 11 | Not having experience of travelling by small vehicle made experience in developing symptoms? | 1. Yes 2. No |  |
| 12 | Travelling with worries for unlawful acts made you experience feeling illness? | 1. Yes 2. No |  |
| 13 | Have you experienced a feeling unwell because of lack of awareness about travel by minibuses? | 1. Yes 2. No |  |
| 14 | Have you developed feeling unwell as a result of role-set? | 1. Yes 2. No |  |
| **Situational associated Variables** | | |  |
| 15 | Could you tell me while you travel in minibuses that made you discomfort? | 1. Minibus Speed Fear 2. Less travel by minibus 3. Working Long Hours Before Travel 4. Road Unsafety 5. Other, specify______________________ |  |
| 16 | Could you tell me the situations that made you develop feeling unwell while you traveled by minibuses? | 1. Regularly travel by minibus 2. Lack of Center for Information about Illness 3. Inability of Suppressing Ride Discomfort 4. Long Distance Travel 5. Lack of Seat Belt Use 6. Other, specify__________________ |  |
| **The Individuals Behaviors** | | |  |
| 17 | Would you tell me your personal behaviours the influence you in experiencing feeling unwell? | 1. Eating Poorly 2. Failing to Physical Exercise before Travel 3. Alcohol Use before Travel 4. Quarrelling in Minibus During Travel 5. Other, specify___________________ |  |

=====================================================
